# Dyslipidaemia and Undernutrition in Children from Impoverished Areas of Maceió, State of Alagoas, Brazil

**DOI:** 10.3390/ijerph7124139

**Published:** 2010-11-30

**Authors:** Gabriela R. S. Veiga, Haroldo S. Ferreira, Ana L. Sawaya, Jairo Calado, Telma M. M. T. Florêncio

**Affiliations:** 1 Faculdade de Nutrição, Universidade Federal de Alagoas, Av. Lorival Melo Mota, Tabuleiro do Martins, 57072-970, Maceió, AL, Brazil; E-Mails: gstux@hotmail.com (G.R.S.V.); haroldo.ufal@gmail.com (H.S.F.); 2 Departamento de Fisiologia da Nutrição, Universidade Federal de São Paulo, Rua Botucatu 862, Vila Clementino, 04023-060, São Paulo, SP, Brazil; E-Mail: alsawaya@unifesp.br; 3 Faculdade de Medicina, Universidade Federal de Alagoas, Campus A.C. Simoes, Tabuleiro do Martins, 57072-970, Maceió, AL, Brazil; E-Mail: jairocalado@terra.com.br

**Keywords:** childhood undernutrition, metabolic disorders, dyslipidemia, anaemia, parasitosis, insulin-like growth factor 1, cortisol, multivariate logistic regression analysis

## Abstract

Chronic undernutrition causes reduced growth and endocrine adaptations in order to maintain basic life processes. In the present study, the biochemical profiles of chronically undernourished children were determined in order to test the hypothesis that chronic undernutrition also causes changes in lipid profile in pre-school children. The study population comprised 80 children aged between 12 and 71 months, including 60 with moderate undernutrition [height-for-age Z (HAZ) scores ≤ −2 and > −3] and 20 with severe undernutrition (HAZ scores ≤ −3). Socioeconomic, demographic and environmental data were obtained by application of a questionnaire, and anthropometric measurements and information relating to sex, age and feeding habits were collected by a trained nutritionist. Blood samples were analysed for haemoglobin, vitamin A, insulin-like growth factor 1 (IGF-1) and serum lipids, while cortisol was assayed in the saliva. Faecal samples were submitted to parasitological investigation. Analysis of variance and χ^2^ methods were employed in order to select the variables that participated in the multivariate logistic regression analysis. The study population was socioeconomically homogeneous, while the lack of a treated water supply was clearly associated with the degree of malnutrition. Most children were parasitised and anaemia was significantly more prevalent among the severely undernourished. Levels of IGF-1 decreased significantly with increasing severity of undernutrition. Lipid analysis revealed that almost all of the children had dyslipidemia, while low levels of high-density lipoprotein were associated with the degree of undernutrition. It is concluded that chronic malnutrition causes endocrine changes that give rise to alterations in the metabolic profile of pre-school children.

## 1. Introduction

The World Health Organization defines undernutrition as the outcome of a variety of pathological conditions that result from deficiencies in absorption, transportation or utilization of nutrients by cells of the body. This type of nutritional disorder mainly affects infants and children of pre-school age [1]. It is estimated that more than one quarter of the global population of children are currently affected by protein-energy undernutrition (PEU), and that 148 million (26.7%) of the 555 million pre-school children living in developing countries are underweight for their age, while 180 million (32.5%) exhibit low height for their age [2,3].

In Brazil, the prevalence of undernutrition was reduced by half during the period 1996–2007 [4] but, owing to social inequality, the condition remains a public health problem, especially in the northern and north-eastern regions of the country [5,6]. According to a nutritional survey conducted in 2005 in the semiarid region of Brazil, the prevalence of undernutrition (stunting) among children living in the State of Alagoas was greater than in other north-eastern states. High amongst the factors influencing this situation was the level of absolute poverty in Alagoas, which remains the highest of any state in the country [7,8].

Undernutrition in children is often accompanied by vitamin and mineral deficits. Indeed, ion deficiency anaemia (sideropenic anaemia) is the most frequent nutritional deficiency worldwide, closely followed by hypovitaminosis [9]. Moreover, in addition to intestinal parasitism, inadequate food intake is considered an important factor in the aetiology of nutritional insufficiency [10]. Chronic undernutrition in early life is associated with diminished intellectual capacity, poor scholastic performance, reduced physical capacity and alterations in metabolic functions, particularly those relating to endocrine processes. Such adaptations tend to diminish the utilization of nutritional substrates in order to maintain the basic life processes, with a concomitant reduction in resources required for normal growth [11,12].

Undernourished children typically present high levels of growth hormone (GH), but low plasma levels of insulin-like growth factor 1 (IGF-1), and this is the main cause of growth deceleration [13]. Furthermore, undernourished children exhibit high levels of cortisol, which promotes protein catabolism and, consequently, delays linear growth [14]. The respiratory quotient (RQ) values in children with chronic undernutrition are larger in comparison with those of eutrophic children, and this represents a further strategy to save energy by reducing lipid oxidation, thus explaining the association between PEU and the accumulation of fat in the liver [11].

The first indication that severe undernutrition could cause hepatic lesions and alterations in the lipid profile was reported by Snapper [15] in 1965 following studies on adults who had experienced interuterine undernutrition during World War II. The relationship between pre-natal exposure to undernutrition and alterations in the lipid profile of adults has since been confirmed in a number of investigations carried out in developed countries [16–18]. However, studies concerning the possible association between hepatic alterations and chronic undernutrition in childhood are scarce and dated [19–21]. In the present study, the biochemical profiles of chronically undernourished children submitted to a semi-confined regime of nutritional rehabilitation in the Centre for Rehabilitation and Nutritional Education of the State of Alagoas (CREN/AL) have been determined in order to test the hypothesis that chronic undernutrition causes changes in lipid profile in pre-school children.

## 2. Methods

Details of the study were submitted to and approved by the Committee of Ethics in Research of the Universidade Federal de Alagoas (protocol no. 009580/2007-26). Written informed consent was obtained from the parents or legal guardians of participating children prior to the commencement of the study.

### 2.1. Study Population

The transversal study involved 80 children in the age range 12 to 71 months who had been enrolled at CREN/AL. All children diagnosed with moderate to severe stature deficit, defined as height-for-age Z (HAZ) scores ≤ −2, were considered for inclusion in the study. Stunted children presenting genetic disorders related to short stature, or chronic diseases such as AIDS, neuropathies, diabetes, cardiopathies and secondary undernutrition, were excluded from the study. Children that were positively diagnosed with pathological conditions during the study were appropriately treated.

### 2.2. Data Collection

Socioeconomic, demographic and environmental data were obtained from the parents or legal guardians of participating children by application of a specific questionnaire that had been previously tested. Anthropometric measurements and information relating to sex, age and feeding habits were obtained by a trained nutritionist using a pre-established protocol. Samples of blood, saliva and faeces were collected from the participating children, stored under cool conditions, and dispatched to an accredited clinical laboratory (Laboratório Nabuco Lopes, Maceió, AL, Brazil) for haematological, biochemical and parasitological assay.

### 2.3. Anthropometric Measurements

The body weight of infants under 24 months of age was determined using Filizola (Campo Grande, MS, Brazil) BP Baby digital scales with a maximum tare of 15 kg and a precision of 5 g. The stature of these children was determined with the aid of an infantometer comprising a non-extendable 105 cm measuring tape graduated in 0.1 cm divisions. For children of 24 months and older, weight was measured using Filizola Personal digital scales with a maximum tare of 150 kg and a precision of 100 g, while stature was determined using a stadiometer comprising a non-extendable 2 m measuring tape graduated in 0.1 cm divisions. In all cases, the scales were tared prior to the measurements, and all procedures were conducted in the presence of mothers or guardians with the children wearing light clothes and with bare feet [22]. Children were classified with regard to nutritional status on the basis of HAZ scores calculated with the aid of World Health Organization (WHO) Anthro software (version 3.0.1, 2007). For children older than 60 months, the 2006 WHO standard tables and formulae [23] were applied to obtain the standard deviation of the indices according to age and sex. Eutrophic children would have HAZ values between +2 and −2 SD, whilst those with HAZ < −2 SD were classified as moderately undernourished and those with HAZ < −3 SD were considered to be severely undernourished.

### 2.4. Food Intake

Participating children remained at CREN/AL from 08:00 h to 17:00 h daily and were offered five meals per day during this period. In order to establish feeding patterns, the average weekly ingestion of nutrients was calculated using Avanutri software (Avanutri Informática, Rio de Janeiro, RJ, Brazil). The prevalence of adequate ingestion of nutrients was determined according to the Dietary Reference Intakes (DRI) published by the National Academy of Sciences—Institute of Medicine [24]. Adequate ingestion of energy was estimated from the published recommendation of 100 kcal/kg weight for nutritional rehabilitation of undernourished children [25]. Adequate ingestion of macronutrients was appraised on the basis of acceptable macronutrient distribution ranges (AMDR) [24]. In order to assess the adequate ingestion of micronutrients (vitamins A, iron, and zinc), the estimated average requirement (EAR) values and the respective cut-off points were employed: when such values were unavailable the adequate intake (AI) values were used [24]. These specific micronutrients were targeted because they are directly related to the growth of infants [26].

### 2.5. Haematological, Biochemical and Parasitological Evaluation

Twelve-hour fasting blood samples (10 mL) were collected via venous puncture and placed in appropriate vials for the separation of serum or plasma as required for biochemical analysis. A complete haemogram (erythrocyte and leukocyte counts, peripheral blood smears) was performed with the purpose of detecting ion-deficiency anaemia. Children presenting concentrations of haemoglobin <11 g/dL (<60 months old) or <11.5 g/dL (60–72 months old) were classified as suffering from ion-deficiency anaemia according to the standards proposed by WHO [27]. The concentration of serum vitamin A (retinol) was determined using high pressure liquid chromatography [28], and children presenting levels below <20 μg/dL were classified as suffering from hypovitaminosis A as recommended by WHO [29]. The concentrations of cholesterol, triglycerides and high-density lipoprotein (HDL) were determined using enzymatic colorimetric methods, while the levels of low-density lipoprotein (LDL) were calculated according to a standard procedure [30]. Evaluation of the lipid profile was performed according to recommendations published by the Sociedade Brasileira de Cardiologia [31]. IGF-1 was determined using a chemiluminescence immunoassay, and children were classified according to the Diagnostic Laboratory System, which specifies minimum and maximum values according to age [32].

Cortisol concentrations were determined in saliva samples obtained from 12-h fasted children on a day separate from that of blood sample collection in order to avoid the influence of stress on the results. Following arrival at CREN/AL (typically at 07:00 h), children were rested for up to 1 h before being required to chew for 2–3 min on the cotton roll of a Salivette kit (Sarstedt, Nümbrecht, Germany; catalogue number 51.1534). The quantitative determination of cortisol was carried out using a radioimmunoassay [33], and values between 4 and 28 nmol/L were considered normal for saliva collected between 07:00 h and 08:00 h for the age group concerned [34].

Plastic recipients, together with appropriate instructions, were provided to mothers for the collection of faecal samples from their children. Parasitological analysis of faecal material was performed according to the Baermann-Moraes method following staining with Wheatley’s trichrome stain [35].

### 2.6. Statistical Analysis

Analysis of variance (ANOVA) and χ^2^ methods were employed in order to select the variables that participated in the multivariate logistic regression analysis. Continuous variables were evaluated by ANOVA in order to convert them into dichotomous variables through the determination of cut-off points. Qualitative variables that were not originally dichotomous were categorised according to the simple frequency distribution. All variables presenting ρ ≤ 0.20 were included in the multivariate analysis. The final logistic regression model was generated by application of the Forward:Wald method with the aid of SPSS software (version 15).

## 3. Results

One child of the original 80 selected moved out of the area and, hence, failed to complete the study. Of the remaining 79 children, 59 (75%) were classified as moderately undernourished (HAZ values in the range −2.01 to −2.94) and 20 (25%) were severely undernourished (HAZ between −3.0 and −4.41). The diet supplied by CREN/AL, which had been adapted to the requirements for rehabilitation, complied with the DRI standards [24] and provided an average total caloric value of 1,250 kcal and 40.2 g of protein to both the moderately and severely undernourished groups [25] (Table 1).

The socioeconomic conditions of the families were homogenous (Table 2) in that their incomes were low, their living conditions were generally precarious (*i.e.*, overcrowded brick dwellings with no floor covering, and reliance on an untreated water supply and septic tank drainage), while most mothers had little schooling and undertook no paid employment. The likelihood of children being severely undernourished increased significantly in families with numerous (7–12) children (Table 2).

Most children presented intestinal parasitism but no statistical differences could be detected between the two groups in this respect (Table 3). The overall prevalence of iron-deficient anaemia was 44.3%, although the proportion of anaemic children was significantly greater within the severely undernourished group than for those presenting moderate undernutrition. Hypovitaminosis A was not detected in children of either group. There were no differences between the groups regarding the levels of salivary cortisol, with the majority of children presenting normal concentrations of this hormone. With regard to IGF-1, however, the levels decreased significantly as the degree of undernutrition increased (Table 3).

Lipid profile analysis demonstrated that 98.9% of the children presented dyslipidaemia. Additionally, levels of HDL were statistically correlated with the degree of undernutrition, such that the likelihood of occurrence of low levels of HDL in children with moderate undernutrition was smaller than for those with severe undernutrition. According to the results of the multivariate analysis (Table 4), the variables most strongly associated with severe undernutrition were untreated water supply, anaemia, low HDL and low IGF-1.

## 4. Discussion

Similar to previous reports [12,36], the present study demonstrated that poor socioeconomic conditions, but most especially reliance on an untreated water supply, strongly influence the development of PEU. Indeed, the chances of children becoming severely undernourished increases by some 600-fold in families that do not treat their drinking water. It is noteworthy, however, that no cases of hypovitaminosis A were detected in the studied population, a finding that contrasts with the results of a previous investigation involving a low-income population [37]. This difference may be explained by the fact that the children in the present study were registered in the National Program of Supplementation of Vitamin A, sponsored by the Brazilian Ministry of Health [38], as confirmed by notes on the vaccination cards of the participants. In contrast, however, the prevalence of iron-deficient anaemia among the studied population was relatively high even though the diet supplied by CREN/AL was perfectly adequate with regard to this mineral. It is assumed that the occurrence of anaemia in infancy derives from the combination of an exceptionally high requirement for iron, imposed by growth on a mineral-poor diet, and the high frequency of infections and parasitic diseases [39,40]. In the present case, the high prevalence of parasitism (81%) together with chronic undernutrition may have contributed to the high prevalence of anaemia detected, reflecting a situation that has been previously observed in a poor population in Maceió [10].

Undernutrition causes great metabolic stress to the body, which typically manifests as an increase in cortisol and a reduction in the resting metabolic rate (RMR) in order to conserve energy [11,12]. In contrast to previous studies [11,41], however, the levels of cortisol observed in the studied population were found to be normal, most likely because the children had already received dietetic treatment for 90 days. It is evident that the supply of a diet that is balanced, particularly with respect to protein, is crucial for the recovery of normal levels of cortisol. Regarding the levels of IGF-1, the present study confirms the severity of undernutrition since a reduction in level of this important growth factor gives rise to serious structural deficits in severely undernourished children. Similar findings have been reported previously for children living in impoverished areas of the State of Sao Paulo, Brazil [42,43].

Together with endocrine alterations, PEU produced modifications in the lipid profiles of children that were similar to those reported in adults who had been exposed to intrauterine undernutrition [44]. It has been demonstrated previously that prenatal undernutrition directly influences the development of altered lipid profiles that are more atherogenic in the long term. Such changes are probably caused by the diminution or inactivation of active liver receptors, or by overproduction of very-low density lipoprotein (VLDL) and LDL, or by defects in the expression of lipoprotein lipase [17,18].

Previous studies involving adolescents with stature deficit have revealed that reductions in RMR are compensated by increased RQ and decreased lipid oxidation [45,46]. It is possible, therefore, that the children who participated in the present study may be accumulating abdominal fat and developing an atherogenic lipid profile by virtue of the alterations described herein. Recent reports [47,48] have confirmed the association between deficient GH response and greater visceral adiposity, dyslipidaemia, insulin resistance and increased cardiovascular risk in overweight adolescents. More specifically, one study revealed a positive association between the levels of plasma IGF-1 and those of HDL-cholesterol in non-diabetic adults [49].

In the present study, dyslipidaemia was characterised by the predominance of low HDL levels (86.1% of the children) together with hypertriglyceridemia. Indeed, both conditions are correlated, although low HDL is caused by an acceleration in catabolism and not by a decrease in the synthesis of these particles [50]. In order to explain the relationship between hypertriglyceridemia and low HDL, two main hypotheses have been put forward, namely: (i) the reduced activity of lipoprotein lipase may hamper the maturation of HDL particles, and (ii) the increased activity of the protein that promotes the transfer of cholesteryl esters from HDL to triglyceride-rich lipoproteins may reduce the levels of HDL particles [51]. Either of these hypotheses could explain the high prevalence (49.4%) of low HDL concomitant with hypertriglyceridemia in the studied population. It is assumed, therefore, that the high frequency of dyslipidaemia observed in the present study was a consequence of the adaptation to chronic undernutrition, although there are currently no studies to support this supposition. Future monitoring of the treated children is, therefore, of vital importance in order to establish if the adequate diet provided by CREN/AL allows recovery of nutritional status and reversal of the atherogenic lipid profile.

## 5. Conclusions

The chronic undernutrition experienced by the study population resulted in endocrine adaptations that gave rise to modifications in lipid profile.

## Figures and Tables

**Table 1:** Anthropometric characteristics and food intake of the study population.

Parameters	Moderately undernourished *n* = 59	Severely undernourished *n* = 20	ρ^a^
**Anthropometric characteristics**			
Boys/girls (*n*)	37/22	9/11	0.165
Age (months; mean ± standard deviation)	43.9 ± 16.5	39.1 ± 16.7	
Weight ((kg; mean ± standard deviation)	14.0 ± 2.5	11.3 ± 3.2	**0.0002**
Height (cm; mean ± standard deviation)	93.5 ± 8.8	83.4 ± 9.3	**0.000**
Height-for-age z score (mean ± standard deviation)	−2.45 ± 0.31	−3.56 ± 0.48	**0.000**
**Dietary intake of nutrients**^b^			
Total caloric value (% of reference value)	100.5	100.2	
Protein (% of reference value)	102.0	101.0	
Carbohydrate (% of reference value)	108.13	105.7	
Lipids (% of reference value)	104.92	102.61	
Zinc (% of reference value)	103.0	105.0	
Vitamin A (% of reference value)	114.69	110.0	
Iron (% of reference value)	129.17	125.8	

a Evaluated using the χ^2^ test.

b Reference values: total caloric value = 100 kcal/kg; protein = 3.5 g/kg; carbohydrate = 55% of the total caloric value; lipids = 25% of the total caloric value; zinc = 4 mg/day; vitamin A = 350 μg/day; iron = 8.5 mg/day.

**Table 2:** Socioeconomic characteristics of the study population.

Parameters	Moderately undernourished		Severely undernourished		ρ^a^
	*n*	%	*n*	%	
**Housing**					
Brick dwelling	41	69.49	17	85.00	0.175
Other	18	30.51	3	15.00	
**Number of rooms**					
1 – 3	36	61.01	13	65.00	0.751
4 – 6	23	38.99	7	35.00	
**Floor covering**					
Yes	21	35.59	4	20.00	0.195
No	38	64.41	16	80.00	
**Sewage conditions**					
Septic tank	47	79.66	16	80.00	0.974
Other	12	20.34	4	20.00	
**Family members**					
1–6	41	69.49	10	50.00	0.316
7–12	18	30.51	10	50.00	
**Rubbish collection**					
Public utility	43	72.88	16	80.00	0.527
Other	16	27.12	4	20.00	
**Water supply**					
Public utility	5	8.47	0	0	0.22^b^
Well	54	91.53	20	100.00	
**Treated water**					
Yes	17	28.81	2	10.00	0.076
No	42	71.19	18	90.00	
**Maternal schooling**					
≤4 years	44	74.58	16	80.00	0.436
>4 years	15	25.42	4	20.00	
**Number of children**					
1–6	49	83.05	12	60.00	**0.038**
7–12	10	16.95	8	40.00	
**Mother occupation**					
With paid employment	4	6.78	3	15.00	0.243^b^
Without paid employment	55	93.22	17	85.00	
**Family income**^c^					
≤1 minimum salary	46	77.97	15	75.00	0.503^b^
>1 minimum salary	13	22.03	5	25.00	

a Evaluated using the χ^2^ test except where indicated otherwise.

b Evaluated using the Fisher test.

c Minimum salary taken as US$ 234.46 per month at the time of the study.

**Table 3:** Haematological, parasitological and biochemical status of the study population.

Variables	Moderately undernourished		Severely undernourished		ρ
	*n*	%	*n*	%	
**Parasitism**					
Positive	51	86.5	18	90.0	0.498^a^
Negative	8	13.5	2	10.0	
**Anaemia**^b^					
Yes	20	33.9	15	75.0	**0.001**^a^
No	39	66.1	5	25.0	
**Hypovitaminosis A**^c^					
Yes	0	0	0	0	
No	59	100	20	20	

Variables	Moderately undernourished	Severely undernourished	ρ
	Mean value ± standard deviation	Mean value ± standard deviation	
**Hormones**^d^			
Cortisol (nmol/L)	7.74 ± 5.92	9.32 ± 8.68	0.400^e^
IGF-1 (ng/mL)	83.8 ± 38.97	60.73 ± 22.19	**0.018**^f^
**Lipid profile**^g^			
Cholesterol (mg/dL)	164.36 ± 32.75	153.05 ± 32.17	0.184^e^
HDL (mg/dL)	36.23 ± 7.59	31.5 ± 7.01	**0.015**^e^
Triglycerides (mg/dL)	109.10 ± 46.46	108.0 ± 29.53	0.921^e^
LDL (mg/dL)	106.28 ± 31.21	99.92 ± 32.42	0.438^e^

a Evaluated using the χ^2^ test.

b Reference value for haemoglobin > 11 g/dL

c Reference value for vitamin A > 20 μg/dL

d Reference values: cortisol = 4–28 nmol/L; IGF-1 = 49–327 ng/mL

e Determined from ANOVA

f Evaluated using the Kruskal Wallis test

g Reference values: cholesterol < 150 mg/dL; HDL ≥ 45 mg/dL; triglycerides and LDL < 100 mg/dL

**Table 4:** Association between the degree of undernutrition and the significant variables as established by logistic regression analysis using the Forward: Wald model.

Variables	Moderate undernutrition	Severe undernutrition	ρ	Odds Ratio	Confidence interval 95%
Absence of treated water	42	18	**0.036**	6.783	1.13–40.51
Presence of anaemia	20	15	**0.014**	0.197	0.05–0.72
Low HDL	50	18	**0.031**	5.189	1.15–23.24
Low IGF-1	12	7	**0.023**	1.029	1.0–1.05
